# How Important Is Protein Diffusion in Prokaryotes?

**DOI:** 10.3389/fmolb.2018.00093

**Published:** 2018-11-13

**Authors:** Paul E. Schavemaker, Arnold J. Boersma, Bert Poolman

**Affiliations:** Department of Biochemistry, University of Groningen, Groningen, Netherlands

**Keywords:** protein diffusion, crowding, prokaryote, reaction rate, diffusion limitation

## Abstract

That diffusion is important for the proper functioning of cells is without question. The extent to which the diffusion coefficient is important is explored here for prokaryotic cells. We discuss the principles of diffusion focusing on diffusion-limited reactions, summarize the known values for diffusion coefficients in prokaryotes and in *in vitro* model systems, and explain a number of cases where diffusion coefficients are either limiting for reaction rates or necessary for the existence of phenomena. We suggest a number of areas that need further study including expanding the range of organism growth temperatures, direct measurements of diffusion limitation, expanding the range of cell sizes, diffusion limitation for membrane proteins, and taking into account cellular context when assessing the possibility of diffusion limitation.

## Introduction

In a cell everything moves and interactions between (macro) molecules are dynamic! This is one of the foremost facts about cells that any student of biochemistry and biology should know. The moving around of components allows proteins and cells to sample different states and provides meaning to the concept of entropy. The molecules move around without the need of work, which is called diffusion. Other types of motion that occur within cells, for example protein transport over membranes, do require work. Diffusion allows molecules to find one another in a cell: substrates need to find enzymes, transcription factors need to find sites on the DNA, membrane proteins need to find the membrane, etc. Thus, it is clear that diffusion is essential, but it is less clear to what degree diffusion is important, which is what we will discuss here. We focus on translational diffusion, which is the displacement of the center of mass of an object, while rotational diffusion is the rotation of an object around its center of mass. We will first discuss general principles of diffusion, including diffusion limitation of reactions and the effect of the intracellular environment on diffusion coefficients; we will provide a summary of experimentally determined protein diffusion coefficients in prokaryotes, and give examples of the consequences of these diffusion coefficients for the cell. Finally, we suggest some principles and experimental directions for further study. We conclude that the importance of protein diffusion coefficients should be assessed in the context of the various layers of complexity in the cell, and that protein diffusion plays, in some instances, a determining role in the physiology and biochemical organization of the cell.

## Overview of diffusion rates and their consequences in prokaryotes

### General principles of diffusion

**Figure 1:**
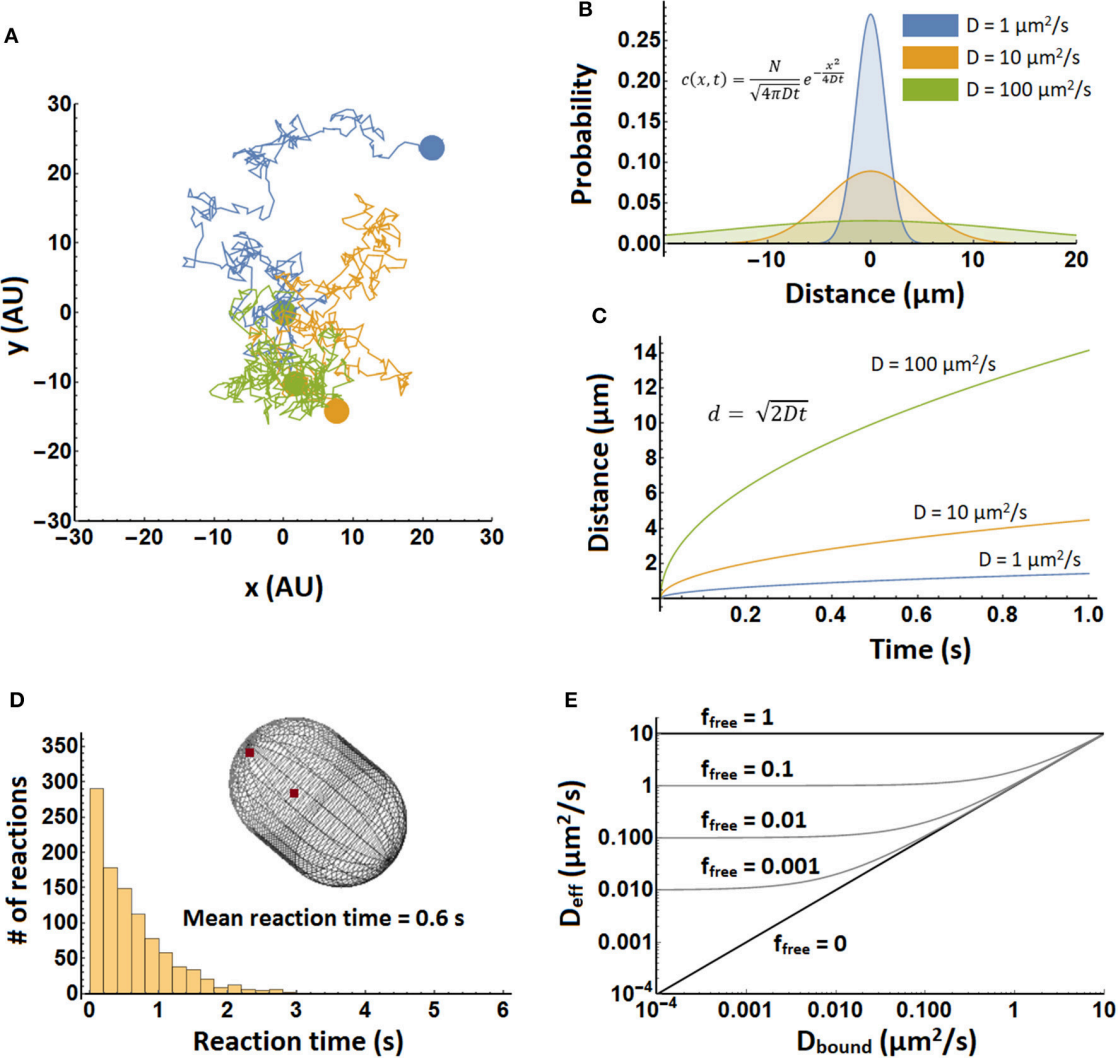
Illustration of diffusion principles**. (A)** Three molecules, each in a different color, undergoing a random walk in two dimensions. Each trajectory consists of 400 steps, and beginning and end are indicated by colored spheres. All three molecules started at position (0,0). **(B)** Probability density in one dimension for the position of a particle after 1 s. Shown are densities for three different diffusion coefficients, which are computed with equation 2. **(C)** The mean distance of a molecule in time. Shown for three different diffusion coefficients. Computed from equation 3 with the number of dimensions set to one. **(D)** Simulation of biomolecular reaction times in a spherocylinder of 1.5 μm in length and 1 μm in width. In 1,000 separate simulations two particles were positioned randomly in the spherocylinder “cytoplasm” and allowed to diffuse with a diffusion coefficient of 10 μm^2^/s and react with a *k*_*on*_ of 10^9^ M^−1^s^−1^. Simulations were performed in Smoldyn (Andrews et al., 2010). **(E)** The effective diffusion coefficient of a complex forming protein as a function of bound diffusion coefficient and free fraction. It was plotted using equation 5, with D_free_ = 10 μm^2^/s. Note that upon binding the free protein takes on the diffusion coefficient of the object it binds to. This means that the top right corner of the graph is somewhat inaccurate.

Perpetual collisions cause molecules to move around inside of cells. Following the behavior of such a molecule, by noting its position every so often, reveals that the direction of travel of a molecule is random (barring structural asymmetries in the medium surrounding the molecule), and the trajectory is that of a random walk (Figure 1A). On the other hand, the step-size of the molecule in each time interval is not random. The step size is determined by the size of the molecule, its interactions with the solvent, and the temperature. The step size (or, more accurately, the step size distribution) is captured by a single parameter, the diffusion coefficient (D). The diffusion coefficient can however also be dependent on length scale, which is called anomalous diffusion (Dix and Verkman, 2008).

A distribution of molecules over space and its evolution in time are described by the diffusion equation (Phillips et al., 2009):

(1) $$\frac{\partial c(x,t)}{\partial t}=D\frac{\partial^{2}c(x,t)}{\partial x^{2}}$$

Here *c*(*x, t*) is the concentration of the molecule at position *x* and time *t*. *D* is the diffusion coefficient. This equation describes diffusion in only one dimension. The movement of the particle in one dimension is completely independent of its movement in the other dimensions. One of the solutions of this equation describes how a group of molecules localized to a point spreads out over time (Figure 1B) (Phillips et al., 2009):

(2) $$c\left(x,t\right)=\frac{N}{\sqrt{4\pi Dt}}e^{-\frac{x^{2}}{4Dt}}$$

Here *N* represents the number of molecules. This equation can also be interpreted as the probability that a single molecule of the diffusing species is going to end up at position x after time t, assuming that diffusing particles do not influence each other. (Note that the molecules may influence each other, say by changing local viscosity, but then we are not dealing with normal diffusion anymore.) Taking the weighted mean over the distances in equation 2 and taking into account multiple dimensions (using Pythagoras' theorem) yields:

(3) $$d=\sqrt{2nDt}$$

Here *d* is the distance and n is the number of dimensions considered. See Figure 1C. Equations 2, 3 do not take into account boundaries such as a cell membrane and are therefore most accurate when length scales are short compared to cell size. Over longer length scales it is better to perform a detailed simulation to take into account the dimensions and shape of the cell (see Figure 1D).

For two particles with diffusion coefficients of 10 μm^2^/s to find each other in a 1 μm^3^ cell takes about 1 s. This can easily be calculated from the bimolecular reaction rate equation:

(4) $$rate=k_{on}\left[mol1\right]\left[mol2\right]$$

The diffusion limited on-rate constant, *k*_*on*_, is about 10^9^ M^−1^s^−1^ (calculated with Equation 6, assuming that the two particles are reactive over their entire surface), and the molecule concentrations are about 1 nM for 1 molecule in 1 μm^3^. This yields a rate of 10^−9^ Ms^−1^, so one molecule reacts in 1 s. A similar result is obtained from a simulation of an association reaction (Figure 1D). Here the reaction was carried out in a spherocylinder of 1.5 μm in length and 1 μm in width. Reaction times are distributed between 0 and 3 s, with the mean at 0.6 s.

A protein can stick to slower diffusing components in the cytoplasm, which can reduce its diffusion coefficient. An effective diffusion coefficient can then be calculated as follows (Schavemaker et al., 2017):

(5) $$D_{eff}=f_{free}D_{free}+\left(1-f_{free}\right)D_{bound}$$

Here, *D*_*eff*_ is the effective diffusion coefficient, *f*_*free*_ is the fraction of the protein of interest that is free (unbound) at equilibrium, *D*_*free*_ is the diffusion coefficient of free protein, and *D*_*bound*_ is the diffusion coefficient when it is bound. The results are shown in Figure 1E.

### Diffusion limited reactions

Diffusion coefficients influence reaction rates when reactions are diffusion limited. The rate of an association reaction between two molecules depends on their concentrations and the on-rate constant, *k*_*on*_, as shown in equation (4). When a reaction is diffusion limited every encounter between the two reactant molecules leads to reaction, so that the *k*_*on*_ depends only on the diffusion coefficients of the two proteins. The diffusion-limited *k*_*on*_ is given by equation (6) for two spherical proteins with a completely reactive surface area (Schreiber et al., 2009):

(6) $$k_{on,\;diff}=4\pi (D1+D2)(R1+R2)$$

Here, *D*1 *and D*2 are the diffusion coefficients of proteins with radii R1 and R2. For proteins with a radius of 0.005 μm, for which the diffusion coefficient is ~10 μm^2^/s in the *E. coli* cytoplasm and ~100 μm^2^/s in dilute solution, the *k*_*on,diff*_ is ~10^8^ M^−1^s^−1^ in the cytoplasm and ~10^9^ M^−1^s^−1^ in dilute solution. Because the right side of the equation deals with single molecules and the left side with moles, the right side has units μm^3^s^−1^ and the left side M^−1^s^−1^. You can convert μm^3^/s into M^−1^s^−1^ by dividing by 10^15^ to convert the volume, and then multiplying by Avogadro's number (6 × 10^23^). This equation is valid only for diffusion in 3D and as such it cannot be used for reactions involving membrane proteins. Most proteins are not reactive over their entire surface and more realistic diffusion limited *k*_*on*_ values are 10^5^-10^6^ M^−1^s^−1^ (Schlosshauer and Baker, 2004; Schreiber et al., 2009). Having multiple binding sites on a protein or electrostatic interactions (Schreiber and Fersht, 1993; Wallis et al., 1995; Alsallaq and Zhou, 2008) can however increase the *k*_*on*_ beyond 10^5^-10^6^ M^−1^s^−1^. Hence, the value of the diffusion limit depends on the proteins involved. Note that we here deal with two definitions of diffusion-limited *k*_*on*_. One is the hard limit given by equation 6, which can only be broken by making motion non-diffusive, for instance by electrostatic attraction. In this definition a reaction is diffusion limited when the *k*_*on*_ is according to equation 6 and the whole surface of the molecule is reactive, which is hardly ever (if ever) the case for molecules in a cell. In the second definition of the diffusion limit only part of the surface is reactive. In this case an increase of the rotational diffusion coefficient could cause the limit to be crossed. Here a reaction is said to be diffusion limited when the coming together of proteins is the slowest step in the reaction, i.e., any necessary conformational changes are very fast. Complications arising from these two different definitions can be bypassed by considering diffusion limitation in the context of a cell. Which is what we do next.

In the cell, the diffusion limitation depends on other processes, because the two proteins may not be constantly present and reactive. These processes include protein synthesis, post-translational modifications, release of proteins from other complexes, transport over membranes, or the cycling through conformational states of one of the binding partners.

If two proteins, A and B, are reactive over their entire surface, they will form a complex as soon as they hit. If on the other hand they have small reactive patches on the surface, they will have to hit each other more often to form a complex. In both these cases, a higher collision rate leads to faster complex formation. When protein A cycles through two states however, of which only one is able to form the complex, the magnitude of the diffusion coefficient is less important: We let A spend an average of 10 s in the inactive state and 10 s in the active state, and the average time for A and B to find each other is 1 s. The inactive A is hit by B on average 10 times before it switches to the active state. When protein A finally does switch to the active state, B binds on average in 1 s. This gives a reaction time of 11 s. If B were to diffuse twice as fast this reaction time would only go down to 10.5 s, while if B were to diffuse twice as slow the reaction time would be 12 s. Thus, the diffusion coefficient of B is relatively inconsequential and the reaction is not diffusion limited. This argument only holds if the active period of A is significantly longer than the time for A and B to bump into one another. If the active period of A is 0.5 s instead of 10 s, B has less successful collisions with A, making the reaction-time sensitive to the diffusion coefficient of B, even though the reaction time will be much longer than the time A and B need to find each other. A real world example of the diminished importance of the cytoplasmic diffusion coefficient is the binding of the transcription inhibitor LacI to its DNA target site. In the search process for its proper binding site, LacI first binds the DNA non-specifically and subsequently scans it, which takes ~90% of the search time (Li and Xie, 2011). If the LacI would diffuse much faster through the cytoplasm this could only reduce the total search time down to 90% of the actually measured time.

Another example where diffusion limitation depends on the context is the formation of gradients during catalysis or protein-protein interactions. Diffusion limitation can result in concentration gradients of reactants (Berg and von Hippel, 1985) when a diffusion limited enzymatic reaction depletes its surroundings. Gradients do not form when the “enzyme” is occupied by its “substrate” (for example in a protein-protein interaction), albeit that the site where the enzyme is synthesized could in principle become the sink. The use of the gradient description depends on the biological context and could work well for the process of translation where association between molecules leads to a reaction (Zhang et al., 2010; Klumpp et al., 2013), or when a membrane has the function of a sink (Schulz and Jorgensen, 2001).

### Diffusion limitation depends on the intracellular environment

**Figure 2:**
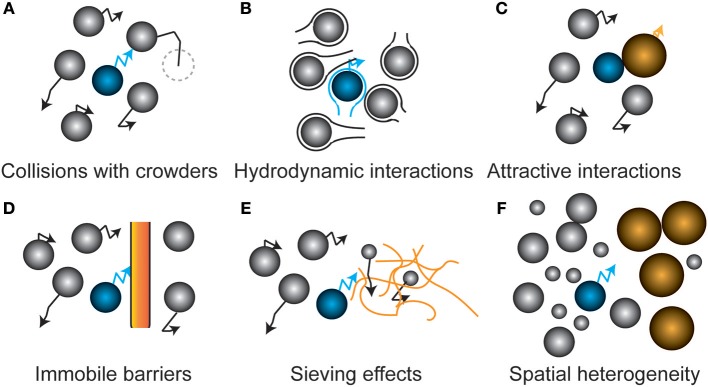
Schematic representation of factors that affect protein diffusion inside cells**. (A)** Hard sphere collisions of the tracer protein (blue) with other freely diffusing proteins (crowders) lowers its diffusion coefficient. **(B)** Movement through the hydrodynamic wake of another protein slows down the tracer protein. At small separation, diffusion increases when another protein moves toward the tracer particle. **(C)** Complex formation with another particle leads to a lower diffusion coefficient due to the increased effective size of the complex. **(D)** Immobile barriers such as membranes confine particles in a given part of the cell. The dimensionality of diffusion is reduced at small distances from the barriers. **(E)** Sieving effects occur when immobile barriers are sieving molecules larger than its pore size, leading to a size-dependent alteration of diffusion. A nucleoid could for example impose this effect on proteins. **(F)** Weak intermolecular forces and steric repulsion between the different biopolymers induces spatial heterogeneity, leading to location-dependent diffusion coefficients of the tracer protein.

How does the intracellular environment determine the diffusion coefficients? The intracellular environment is not a homogeneous medium with a single diffusion coefficient for a given protein; many factors may retard the diffusion of a protein in a crowded cell, increasing the fuzziness of diffusion limits (Figure 2). Moreover, the thermodynamic non-ideality of the cytoplasm makes the diffusion coefficient not simply a sum of its contributors. We will discuss some of the most important contributors below.

The cell is highly crowded with macromolecules that provide steric barriers for a diffusing protein (Figure 2A). The simplest systems in which to study the effect of crowding on diffusion are solutions of a single type of macromolecule at varying concentrations. Such experiments have shown a marked decrease in the diffusion coefficient with concentration (or volume fraction), with the fold changes getting bigger at higher concentrations (Tokuyama et al., 2011). As for the magnitude of the decrease we will compare the relative diffusion coefficients (D/D_0_) at a volume fraction of 0.2, which is similar to what has been found for the *E. coli* cytoplasm (Konopka et al., 2009). In some cases data is expressed in g/L and we have converted the values by using the protein specific volume. From this we find that D/D_0_ is 0.3 for Barstar (10 kDa) (Nesmelova et al., 2002), 0.14 for SH3 (10 kDa) (Rothe et al., 2016), 0.41 for Myoglobin (16 kDa) (Nesmelova et al., 2002), 0.25 for Hemoglobin (*Homo sapiens*, 64 kDa) (Keller et al., 1971), 0.14 for BSA (66 KDa) (Nesmelova et al., 2002), and finally 0.1 for Hemoglobin (*Lumbricus terrestris*, 3700 kDa) (Gros, 1978). This limited set of data shows a decrease in the relative diffusion coefficient with molecular weight, but also a rather large spread in relative diffusion coefficient at each molecular weight. This decrease in relative diffusion coefficient with molecular weight is also seen in the *E. coli* cytoplasm (see below). However, the relative diffusion coefficient is bigger in the *in vitro* systems (average of 0.2 or higher for proteins of 10–66 kDa) than in the *E. coli* cytoplasm (average < 0.1 from 27–1,000 kDa). This difference may be related to a difference in the surface properties between proteins that are often used in *in vitro* experiments and those that predominate the cytoplasm. This could be a reduced binding affinity between the proteins, after all most proteins cannot be concentrated to the level needed for these *in vitro* diffusion measurements. These studies on single macromolecule solutions are simple but they have the limitation that you always change both the background macromolecules and the one you measure the diffusion coefficient of. This complicates interpretations of molecular weight vs. diffusion coefficient data. The diffusion coefficient of hemoglobin(CO), myoglobin(CN), BSA, and aldolase in a background of aldolase, BSA, ovalbumin, or ribonuclease also shows a bigger drop in diffusion coefficient for bigger proteins, but here also the trend is not perfect (Muramatsu and Minton, 1988). This finding is contradicted by a study on the diffusion coefficients of rhodamine green, dextran, differently-sized DNAs, albumin, and differently-sized nanospheres in the presence of the artificial crowder Ficoll 70. Here, the fold drop in diffusion coefficient is similar for objects of varying sizes (Dauty and Verkman, 2004). Likely, the use of a polymer crowder instead of a protein crowder induces different behavior. Concentrated polymers entangle and their effects are dictated by the monomer concentration and not their size, in contrast to strictly globular proteins. Also, the objects tested are of a rather different nature making interpretation difficult. That the nature of the macromolecule matters for its diffusion coefficient, and its response to crowding, is shown in a study on the intrinsically disordered protein α-synuclein. The diffusion coefficient (determined by NMR) of α-synuclein is slower than that of chymotrypsin inhibitor 2 under dilute conditions, but is faster in the presence of crowders (BSA, lysozyme, Ficoll 70, or PVP) (Wang et al., 2012).

Another steric effect is the presence of immobile barriers that preclude the long distance movement of a molecule (Figure 2D). For example, proteins in the periplasm experience confinement from the cytoplasmic and outer membrane. The nucleoid can become a barrier for larger, ribosome-sized, proteins or protein-aggregates that are too large to diffuse through the meshwork presented by the DNA (Figure 2E) (Bakshi et al., 2012). In a model of LacI diffusion in the nucleoid it was concluded that DNA dynamics determines the motion of LacI to a large extent (Chow and Skolnick, 2017). During an osmotic upshift, next to the increased crowding, invaginations provide temporal barriers for proteins (Mika et al., 2010) until the cell recovers its volume and resumes growth. Small organic molecules experience much less reduction in their diffusion, which allows nutrients and compatible solutes to probe the entirety of the cell even under extreme conditions of osmotic stress and aid the recovery from osmotic stress (Mika et al., 2010). Another example of immobile barriers that depend on the physiological state of the cell occurs during energy depletion where a decrease in diffusion occurs only for very large particles, i.e., viral nanoparticles, plasmids, and aggregates. Here, the cytoplasm of *E. coli* and yeast transits to a colloidal-glassy or gel-like state that sieves larger particles (Parry et al., 2014; Joyner et al., 2016; Munder et al., 2016). In eukaryotes membrane proteins appear to encounter barriers to diffusion such as cytoskeletal elements that temporarily confine them (Saxton and Jacobson, 1997). The consequences of such confinement for (apparent) diffusion coefficients has been studied theoretically (Saxton, 1995). It is unclear how well these findings can be transferred to prokaryotes but it appears that at least some outer membrane proteins in *E. coli* are confined (Table 1).

**Table 1:** Overview of experimentally determined diffusion coefficients.

**Molecule**	**Organism**	**Diffusion coefficient (D; μm^2^/s)**	**Comments**	**References**
NBD-glucose	*Escherichia coli*	50	0.423 kDa	Mika et al., 2010
GFP	Dilute solution	87	27 kDa	Potma et al., 2001
GFP	*Dictyostelium discoideum*	24	Cytoplasm, 27 kDa	Potma et al., 2001
GFP	*Mus musculus*	27	Fibroblast cytoplasm, 27 kDa	Swaminathan et al., 1997
GFP	*Escherichia coli*	3-14	Cytoplasm, 27 kDa	Konopka et al., 2009; Mika and Poolman, 2011
GFP	*Lactococcus lactis*	7	Cytoplasm, 27 kDa	Mika et al., 2014
GFP	*Bacillus subtilis*	>1	Cytoplasm, germinated spores, 27 kDa	Cowan et al., 2003
GFP	*Bacillus subtilis*	~0.0001	Spore cytoplasm, 27 kDa	Cowan et al., 2003
GFP	*Caulobacter crescentus*	8	Cytoplasm, 27 kDa	Llopis et al., 2012
GFP	*Haloferax volcanii*	5.5	Cytoplasm, 27 kDa	Schavemaker et al., 2017
mCherry	*Pseudomonas aeruginosa*	4	Cytoplasm, 27 kDa	Guillon et al., 2013
TorA-GFP	*Escherichia coli*	9	Cytoplasm, 30 kDa, in ΔtatABCDE strain	Mullineaux et al., 2006
PtsH-YFP	*Escherichia coli*	3.8	Cytoplasm, 36 kDa, some degradation of the protein	Kumar et al., 2010
CheY-GFP	*Escherichia coli*	4.6	Cytoplasm, 41 kDa	Cluzel et al., 2000
Crr-YFP	*Escherichia coli*	2.0	Cytoplasm, 45 kDa, some degradation of the protein	Kumar et al., 2010
NlpA_noLB_-GFP	*Escherichia coli*	2.7	Cytoplasm, 55 kDa	Nenninger et al., 2010
TorA-GFP2	*Escherichia coli*	8.3	Cytoplasm, 57 kDa, 2x GFP in tandem	Nenninger et al., 2010
AmiA_noSP_-GFP	*Escherichia coli*	7.1	Cytoplasm, 58 kDa	Nenninger et al., 2010
CFP-CheW-YFP	*Escherichia coli*	1.5	Cytoplasm, 72 kDa, some degradation of the protein	Kumar et al., 2010
MBP-GFP	*Escherichia coli*	2.5	Cytoplasm, 72 kDa	Elowitz et al., 1999
torA-GFP3	*Escherichia coli*	6.3	Cytoplasm, 84 kDa, 3x GFP in tandem	Nenninger et al., 2010
CFP-CheR-YFP	*Escherichia coli*	1.7	Cytoplasm, 86 kDa, some degradation of the protein	Kumar et al., 2010
DnaK-YFP	*Escherichia coli*	0.67	Cytoplasm, 96 kDa, some degradation of the protein	Kumar et al., 2010
torA-GFP4	*Escherichia coli*	5.5	Cytoplasm, 111 kDa, 4x GFP in tandem	Nenninger et al., 2010
torA-GFP5	*Escherichia coli*	2.8	Cytoplasm, 138 kDa, 5x GFP in tandem	Nenninger et al., 2010
HtpG-YFP	*Escherichia coli*	1.7	Cytoplasm, dimer of 198 kDa	Kumar et al., 2010
CFP-CheA-YFP	*Escherichia coli*	0.44	Cytoplasm, 250 kDa, some degradation of the protein	Kumar et al., 2010
LacI-Venus	*Escherichia coli*	3	Cytoplasm, tetramer of ~260 kDa, freely diffusing, when DNA binding is included D = 0.4 μm^2^/s	Elf et al., 2007
β-galactosidase	Dilute solution	31	Tetramer of 466 kDa	Hahn and Aragon, 2006
β-galactosidase-GFP	*Escherichia coli*	0.7	Cytoplasm, tetramer of 582 kDa	Mika et al., 2010
β-galactosidase-GFP	*Lactococcus lactis*	0.8	Cytoplasm, tetramer of 582 kDa	Mika et al., 2014
Ribosome	*Escherichia coli*	0.04	Cytoplasm, fully active, includes all states of translation	Bakshi et al., 2012
Ribosome (free, 30S)	*Escherichia coli*	0.6	Cytoplasm, freely diffusing, 1 MDa	Bakshi et al., 2012
Ribosome (bound)	*Escherichia coli*	0.055	Cytoplasm, bound fraction	Sanamrad et al., 2014
Ribosome (free, 30S or 50S)	*Escherichia coli*	0.4	Cytoplasm, free fraction	Sanamrad et al., 2014
Ribosome (bound)	*Caulobacter crescentus*	0.0002– < 0.0011	Cytoplasm, obtained from model that includes a bound and free fraction	Llopis et al., 2012
Ribosome (free, 50S)	*Caulobacter crescentus*	0.018–0.042	Cytoplasm, obtained from model that includes a bound and free fraction	Llopis et al., 2012
Ribosome (free, 50S)	*Caulobacter crescentus*	0.36–0.39	Cytoplasm, after cells were treated with rifampicin or kasugamycin	Llopis et al., 2012
Carboxysome	*Synechococcus elongatus*	0.000046	Cytoplasm, constrained movement; consists of ~5000 monomers of shell protein and ~2000 monomers of rubisco	Savage et al., 2010
mRNA	*Escherichia coli*	0.001-0.03	Cytoplasm, diffusion is anomalous, mRNA in complex with many copies of MS2-GFP	Golding and Cox, 2004, 2006
DNA	*Escherichia coli*	0.0004-0.0007	Chromosomal loci, apparent *D* as DNA doesn't move freely	Reyes-Lamothe et al., 2008
PvdS-eYFP	*Pseudomonas aeruginosa*	1	Cytoplasm, PvdS is a sigma factor, 48 kDa	Guillon et al., 2013
PvdA-eYFP	*Pseudomonas aeruginosa*	0.5	Cytoplasm, 76 kDa	Guillon et al., 2013
PvdQ-mCherry	*Pseudomonas aeruginosa*	0.2	Periplasm, 111 kDa	Guillon et al., 2013
FpvF-mCherry	*Pseudomonas aeruginosa*	0.2	Periplasm, 59 kDa	Guillon et al., 2013
GFP	*Escherichia coli*	2.6	Periplasm; TorA signal sequence removed upon export to periplasm	Mullineaux et al., 2006
MotB-GFP	*Escherichia coli*	0.0075-0.0088	Plasma membrane, freely diffusing, dimer	Leake et al., 2006
TatA-GFP	*Escherichia coli*	0.13	Plasma membrane	Mullineaux et al., 2006
Tar(1-397)-YFP	*Escherichia coli*	0.22	Plasma membrane, 4 transmembrane helices	Kumar et al., 2010
Tsr(1-218)-YFP	*Escherichia coli*	0.18	Plasma membrane, 4 transmembrane helices	Kumar et al., 2010
LacY-YFP	*Escherichia coli*	0.027	Plasma membrane, 12 transmembrane helices	Kumar et al., 2010
MtlA-YFP	*Escherichia coli*	0.028	Plasma membrane, 12 transmembrane helices	Kumar et al., 2010
Tar-YFP	*Escherichia coli*	0.017	Plasma membrane, 12 transmembrane helices	Kumar et al., 2010
TetA-YFP	*Escherichia coli*	0.09	Plasma membrane, 12 transmembrane helices	Chow et al., 2012, see also discussion in ref. Mika et al., 2014
NagE-YFP	*Escherichia coli*	0.020	Plasma membrane, 16 transmembrane helices	Kumar et al., 2010
FliG-GFP	*Escherichia coli*	0.0049	Attached to flagellum basal body	Fukuoka et al., 2007
BcaP-GFP	*Lactococcus lactis*	0.02	Plasma membrane, 12 transmembrane helices	Mika et al., 2014
LacSΔIIA-GFP	*Lactococcus lactis*	0.02	Plasma membrane, 12 transmembrane helices	Mika et al., 2014
PleC-eYFP	*Caulobacter crescentus*	0.012	Plasma membrane, freely diffusing, 4 transmembrane helices	Deich et al., 2004
Lipopolysaccharide	*Salmonella typhimurium*	0.02	Outer membrane	Schindler et al., 1980
BtuB	*Escherichia coli*	0.05-0.10	Outer membrane, 22-stranded β-barrel, when disconnected from its binding partner TonB D = 0.27 μm^2^/s	Spector et al., 2010
OmpF	*Escherichia coli*	0.006	Outer membrane, trimer, 16-stranded β-barrel, diffusion is restricted to an area with a diameter of 100 nm	Spector et al., 2010
LamB (λ-receptor)	*Escherichia coli*	0.15	Outer membrane, LamB appears to be tethered and diffusion is restricted to area with 50 nm diameter	Oddershede et al., 2002

In addition, non-covalent chemical interactions with the surrounding proteins change the diffusion of a protein (Figure 2C); these interactions can be hydrophobic, van der Waals, electrostatic or hydrogen-bonding, and thus strongly depend on the properties of both the protein and the proteome. Again we look first at the simplest studies of diffusion with single types of protein at different concentrations. Lysozyme changes its affinity for dimerization depending on the pH. When diffusion measurements are performed at a pH that favors the dimer the diffusion coefficient drops faster with lysozyme volume fraction than at a pH that favors the monomer (Nesmelova and Fedotov, 1998). We see similar behavior when more than one protein is in solution. For example, the diffusion coefficients of BSA and aldolase drop much faster with background protein concentration if this background protein is ribonuclease rather than aldolase, BSA, or ovalbumin (Muramatsu and Minton, 1988). The influence of interactions is seen also in a diffusion study of a mixture of SH3 and BSA at varying concentrations. Whereas, these proteins when studied independently follow the Stokes-Einstein relation they fail to do so when in a mixture (Rothe et al., 2016). We are unaware of *in vitro* studies that use more complex mixtures for studying diffusion, with the exception of diffusion measurements on proteins in cell lysates (Wang et al., 2010). The reason for this is presumably that complex mixtures of proteins cannot be concentrated to cellular levels. And even if you could do so you would probably not be able to avoid potential aggregation or phase separations. Computational studies can be carried out on complex mixtures and have the advantage that you can see exactly what is going on. For example, it was possible to study the diffusion of proteins in a simulated *E. coli* cytoplasm that contained the 50 most abundant macromolecules at appropriate concentrations. However, there was no attempt to study the effect of changes in total macromolecule concentration (McGuffee and Elcock, 2010). A limit of such computational studies is the limited time window over which diffusion can be monitored. That interactions between macromolecules affect diffusion behavior *in vivo* is shown in a study of differently charged versions of GFP in the cytoplasm of three prokaryotes, which revealed that positive GFPs can diffuse up to 100-fold slower due to their interactions with ribosomes (Schavemaker et al., 2017).

Hydrodynamic interactions, which are caused by the flow field of other diffusing particles, have been proposed to have strong long-range effects that slow down proteins (Figure 2B), and such interactions are needed to simulate the lower diffusion constants of GFP in the cell (Ando and Skolnick, 2010). Although this phenomenon is well studied in colloidal physics, hydrodynamic interactions of proteins inside cells have only been studied by molecular dynamics simulations.

The above-mentioned associative and repulsive effects act on all the biopolymers in the cell, and likely induce spatial heterogeneity in cells (Figure 2F) (Spitzer and Poolman, 2009, 2013; Yu et al., 2016; van den Berg et al., 2017). Spatial heterogeneity is most pronounced where proteins phase separate from the cytoplasmic pool of proteins; these effects are most often associated with eukaryotic cells, but microdomains also occur in bacteria, for example as inclusion bodies, the nucleoid, or the cell-polarity inducing assemblies in *Caulobacter crescentus* (Perez et al., 2017). Such domains likely alter the mobility of macromolecules in the cell. The rate of assembly of phase separations may be diffusion limited and can give rise to spatial pattern formation in complex reaction-diffusion networks when competing binding partners are present (Saha et al., 2016). Stress conditions may increase spatial heterogeneity, in particular in energy-starved cells. It has been suggested that ATP at physiological concentrations (5–10 mM) acts as a biological hydrotope in cells, preventing phase separation of proteins (Patel et al., 2017). When ATP levels decreased, the disordered proteins increasingly self-associate, giving rise to regions that are more crowded than others are. As we proposed previously, it is likely that small molecules mediate the protein organization, also in cells (Spitzer and Poolman, 2005, 2009; van den Berg et al., 2017). This implies that in the cell there may be physiology-dependent regions with lowered diffusion coefficients for a given protein, and here reactions could become rate limiting.

In summary, the diffusion coefficient and the occurrence of diffusion-limited reactions depend on the location of the protein in the cell, the physiological state of the cell itself, and the size and chemical nature of the diffusing species.

### What are the diffusion coefficients of proteins in cells?

A large set of diffusion coefficients has been determined in prokaryotes, of which we provide a comprehensive overview in Table 1. For comparison we include data on a small molecule in *Escherichia coli*, proteins in some eukaryotes, and proteins in dilute solution. Diffusion coefficients have been measured for proteins in the cytoplasm, plasma membrane, periplasm, and outer membrane. Most diffusion coefficients have been determined for proteins in *E. coli*, but a decent amount of data is also available for the bacteria *Caulobacter crescentus, Pseudomonas aeruginosa*, and *Lactococcus lactis*. Most *in vivo* diffusion coefficients have been determined with fluorescence recovery after photo-bleaching (FRAP), some are determined by single particle tracking (SPT) or fluorescence correlation spectroscopy (FCS). For a short description of these techniques we refer to (Mika and Poolman, 2011). For *in vitro* determinations of diffusion coefficients you can use NMR (Wang et al., 2012), analytical ultracentrifugation (Tyn and Gusek, 1990), the rate of transfer of protein over a porous membrane, or dynamic light scattering (van Holde et al., 2006).

**Figure 3:**
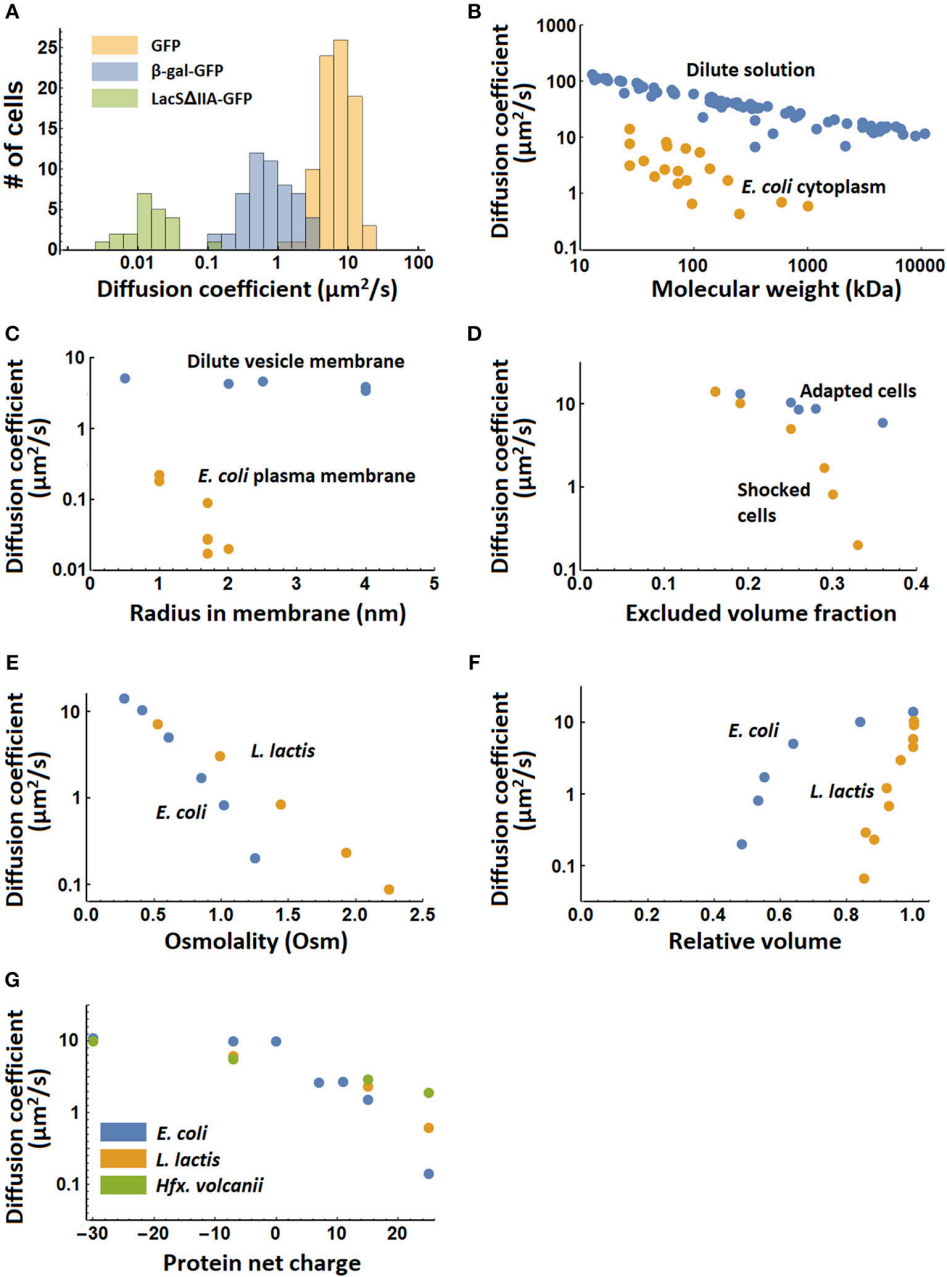
Systematic variation of diffusion coefficients with protein and environment properties. **(A)** Variation of diffusion coefficient within a population of cells for the proteins GFP and β-galactosidase-GFP (tetramer) in the cytoplasm, and LacSΔIIA-GFP in the membrane of *Lactococcus lactis* (Mika et al., 2014). **(B)** The dependence of diffusion coefficient on molecular weight in dilute solution (Tyn and Gusek, 1990) and the *Escherichia coli* cytoplasm (Elowitz et al., 1999; van den Bogaart et al., 2007; Konopka et al., 2009; Kumar et al., 2010; Mika et al., 2010; Nenninger et al., 2010; Bakshi et al., 2012). **(C)** The dependence of diffusion coefficient on radius of the membrane spanning part of membrane proteins in giant unilamellar vesicles (GUVs) (Ramadurai et al., 2009) and in the *Escherichia coli* plasma membrane (Kumar et al., 2010). The radii for the proteins studied in the *E. coli* membrane are calculated from the number of transmembrane helices (Kumar et al., 2010) and the radius of a single helix peptide reported in (Ramadurai et al., 2009). **(D)** The dependence of diffusion coefficient of cytoplasmic GFP on excluded volume fraction in adapted and shocked *Escherichia coli* cells (Konopka et al., 2009). **(E)** The dependence of the diffusion coefficient of cytoplasmic GFP on medium osmolality after osmotic shock for *Escherichia coli* and *Lactococcus lactis* (Konopka et al., 2009; Mika et al., 2014). The growth medium had the same osmolality as the first points on the graph. **(F)** The dependence of the diffusion coefficient of cytoplasmic GFP on the relative cell volume after osmotic shock in *Escherichia coli* and *L. lactis* (Mika et al., 2014). **(G)** The dependence of the cytoplasmic diffusion coefficient of GFP variants on their net charge in *Escherichia coli, Lactococcus lactis* and *Haloferax volcanii* (Schavemaker et al., 2017). There is no data for −30 GFP in *L. lactis*.

The values represented in Table 1 are means or medians over populations of cells. Typically, there is considerable variation in the diffusion coefficient between cells (Konopka et al., 2009; Mika et al., 2014). We illustrate this in Figure 3A where we show histograms of the diffusion coefficients of GFP, a large enzyme complex (β-galactosidase-GFP), and a membrane protein (LacSΔIIA-GFP) in *L. lactis*. In addition, not all diffusive processes can be described by a single diffusion coefficient. In some cases, the molecules are confined (Fukuoka et al., 2007) or exhibit anomalous diffusion (Golding and Cox, 2006).

The diffusion coefficients show differences for the same molecule (GFP or mCherry) in the cytoplasms of *C. crescentus* (8 μm^2^/s), *P. aeruginosa* (4 μm^2^/s), *L. lactis* (7 μm^2^/s), and the archaeon *Hfx. volcanii* (5.5 μm^2^/s), which all fall within the range that is measured for *E. coli* (3–14 μm^2^/s). Hence, it is not clear whether these differences are real and could be due to measurement error, the method used, or different growth and measurement conditions. *E. coli* and *L. lactis* give roughly equivalent diffusion coefficient of β-galactosidase-GFP, as well as for membrane proteins with 12 transmembrane helices. There are differences between *E. coli* and *C. crescentus*, with diffusion coefficients of 0.04 μm^2^/s vs. 0.0002–<0.0011 μm^2^/s for ribosome diffusion, respectively, and 0.18–0.22 μm^2^/s vs. 0.012 μm^2^/s for membrane proteins with 4 transmembrane helices. It is currently not clear what causes the differences in mobility in *E. coli* and *C. crescentus*.

It is not just the isolated values listed in Table 1 that matter, we also need to consider how diffusion values vary in different contexts (*e.g.*, physicochemical state of the cell) and with protein properties (Figures 3B–G). Protein diffusion coefficients go down with molecular weight of the protein, both in dilute solution and in the *E. coli* cytoplasm (Figure 3B). This is also seen for membrane proteins in relation to their (membrane-embedded) radius in giant unilamellar vesicles (GUVs) and the *E. coli* plasma membrane (Figure 3C). Increasing the salt concentration of the outside medium reduces the water content of *E. coli* cells and increases the volume fraction that is excluded by macromolecules. When cells are allowed to adapt to the increased salt concentration, the diffusion coefficient drops less fast with excluded volume fraction than when this is done swiftly (shocked) (Figure 3D). The drop in diffusion coefficient with osmotic shock is less for *L. lactis* than for *E. coli* (Figure 3E), while the drop in diffusion coefficient with relative cell volume (after shock) is much larger in *L. lactis* than in *E. coli* (Figure 3F). The reason for this difference between *L. lactis* and *E. coli* is not clear but could have something to do with different levels of crowding or different co-solvents in the cytoplasm. Finally, the diffusion coefficient of different surface-modified variants of GFP depends on their net charge, with positive proteins diffusing up to a 100-fold slower. This effect is strongest in *E. coli* but is also present in *L. lactis* and the archaeon *Haloferax volcanii* (Figure 3G). *L. lactis* and *H. volcanii* have a (much) higher ionic strength than *E. coli*, which explains the smaller impact of a cationic surface on the slowing of diffusion in these microbes. Thus, “electrostatic lubrication” seems important in keeping the macromolecules moving and may have been a driver in the evolution of the cellular proteomes. In summary, the measured diffusion coefficients depend strongly on the protein size, surface chemistry, as well as the intracellular environment.

### Examples of diffusion limitation in prokaryotes

Lowering the diffusion coefficient of a protein indefinitely would cause any reaction to become diffusion limited. Therefore, a study of diffusion coefficients and diffusion limitation of processes is pertinent. There are not many examples where the importance of the diffusion coefficient actually has been demonstrated. Below, we summarize cases where diffusion limitation appears to occur. Some more discussion of diffusion processes in prokaryotes can be found in (Soh et al., 2010).

#### The on-rate constant of barnase-barstar goes beyond the diffusion limit

The diffusion limited *k*_*on*_ starts at 10^5^-10^6^ M^−1^s^−1^ (Schlosshauer and Baker, 2004; Alsallaq and Zhou, 2008), but protein pairs such as Barnase-Barstar from *Bacillus amyloliquefaciens* manage to have a *k*_*on*_ of 10^8^-10^10^ M^−1^s^−1^ (Schreiber and Fersht, 1993; Wallis et al., 1995; Alsallaq and Zhou, 2008). Barnase is an extracellular ribonuclease that is bound by Barstar in the cytoplasm to prevent damage of endogenous RNA (Buckle et al., 1994). The fact that the reaction is electrostatically steered, and that the on-rate constant is two orders of magnitude higher than the non-electrostatic diffusion limit, suggests that the diffusion coefficient is important for this reaction. (Note that we use the second definition of diffusion limitation as outlined in section Diffusion Limited Reactions.) Another protein pair with a very high on-rate constant is ColicinE9-Im9. ColicinE9 is a secreted toxin with DNase activity. Again, its binding partner, Im9, is used to prevent damage in the cytoplasm where ColicinE9 is made (Wallis et al., 1995). Note that the increase in on-rate constant could be there to make the complex bind more tightly rather than increase the on-rate *per se*. A direct determination of diffusion limitation has not been carried out. As a final qualifier we add that the *k*_*on*_ measurements were carried out on dilute samples and it isn't clear how well these results transfer to the *in vivo* (crowded) situation.

There are several other bacterial proteins that form complexes with high *k*_*on*_ values, although this is not always demonstrated with the physiological binding partner: SecB from *E. coli* was shown to interact with BPTI (Bovine pancreatic trypsin inhibitor) with a *k*_*on*_ of 5 × 10^9^ M^−1^s^−1^ (Fekkes et al., 1995), and the chaperone complex GroEL interacts with various proteins with a high *k*_*on*_ values, including the unfolded state of barnase with a *k*_*on*_ of 0.35–1.8 × 10^8^ M^−1^s^−1^ (Gray and Fersht, 1993; Perrett et al., 1997), MBP (Maltose binding protein) with a *k*_*on*_ of 0.9–7.0 × 10^7^ M^−1^s^−1^ (Sparrer et al., 1996), and DHFR with a *k*_*on*_ of 3 × 10^7^ M^−1^s^−1^ (Clark and Frieden, 1997). It is however not clear whether these GroEL interactions are really diffusion limited because the unfolded proteins provide many more interaction opportunities than folded proteins, so the limit of 10^5^-10^6^ M^−1^s^−1^ may not apply.

#### Translation and cell growth rate are limited by charged tRNA availability

Protein production could limit the growth rate and is set by the number of ribosomes, how fast they can start and end the production of one protein, and how fast they can elongate the proteins. In individual cases protein production can be limited by ribosome binding site strength rather than elongation rate. Elongation consists of the arrival of ternary complex, a complex that consists of amino acyl-tRNA, EF-Tu and GTP, and its processing by the ribosome. Using a computational model of the translation process it was found that if many ribosomes (100) are synthesizing the same protein and thus using the same amino acids, the rate per codon was decreased because of diffusion limitation. The effect was exacerbated when the diffusion coefficient was decreased after simulating an osmotic shock (Zhang et al., 2010). It is not clear whether this diffusion limitation is present at actual cellular conditions and amino acid sequences.

In another study (Klumpp et al., 2013) the rate of translation was also found to be diffusion limited. In the calculations, Michaelis-Menten kinetics was assumed for amino acid incorporation. The K_M_ was calculated under the assumption that the reaction is diffusion limited. They estimated a diffusion coefficient of 1 μm^2^/s for the ternary complex, from which they determine the *k*_*on*_ for binding of ternary complex to ribosome to be 10^7^ M^−1^s^−1^. The rate of going from the ternary complex being bound to the ribosome to amino acid chain elongation, *k*_*elong*_, was set at 30 s^−1^. From this they calculated that the diffusion limited K_M_ is 3 μM, which compares to the concentrations of tRNA in *E. coli*, 3–30 μM. The finding that the concentrations of tRNA are equal or higher than the diffusion limited K_M_ is taken as evidence that the process operates at diffusion limited rate. The estimate of the diffusion limited *k*_*on*_ is made on the condition that Equation 6 is valid, which assumes that the molecules that react can have any orientation upon collision and react immediately. This is unlikely to be the case. Diffusion limited *k*_*on*_'s are also not necessarily single values as electrostatic interactions may steer the interaction and make the reaction faster. Next, they made a model that takes into account allocation of resources to different parts of the proteome. The translation speed is limited by the association rate of the ternary complex to the ribosome, which depends on both *k*_*on*_ and concentration. Allocating resources to increasing the concentration of ternary complex will limit the resources that can be put into ribosome production. The cell growth rate is a function of both translation speed and ribosome concentration, and thus cell growth rate and allocation of resources are influenced by the diffusion coefficient of the ternary complex.

#### The combination of cell size and protein concentration in prokaryotic and eukaryotic cells is optimized to facilitate rapid diffusion

Say you hold the number of proteins in an *E. coli* cell constant but would decrease cell size, then the distances that need to be overcome by diffusion are smaller, but the crowding increase leads to slower diffusion. If you make the cell bigger, the distances become larger but diffusion becomes faster. This scenario has been turned into a quantitative model, which shows that for prokaryotes the cell diameter is predicted to be 1.1 μm, and for eukaryotes 15.7 μm at the smallest characteristic diffusion times (Soh et al., 2013). It is claimed that these diameters are comparable to the typical sizes of the prokaryotic and eukaryotic cells, indicating that the combination of cell size and macromolecule concentration is optimized for rapid diffusion, and that there are diffusion-limited processes in these cells. This prediction of cell size depends on the number of proteins in these cells, which is 3 × 10^6^ for prokaryotes and 8 × 10^9^ for eukaryotes. In the study, it is mentioned that the model provides an argument for determining what the sizes of prokaryotic and eukaryotic cells should be, yet no argument is provided that states why the number of proteins ought to be 3 × 10^6^ and 8 × 10^9^. Furthermore, the characteristic distance that diffusion needs to bridge is taken to be the size of the cell. For many reactions the targets are probably much closer.

### Differences in diffusion coefficients leading to functional differences

We discussed several cases where diffusion could limit rates of other processes in the cell. These consequences of the diffusion coefficients are essentially efficiency improvements; they do not arbitrate on the existence of phenomena. Here we will give two examples in which diffusion makes a functional difference, which are phenomena that would not exist were it not for certain diffusion coefficients.

#### The min system oscillation in *E. coli*

Cell division in *E. coli* creates two equal-sized daughter cells. A key protein in cell division is FtsZ, which forms a ring in the middle of the cell that helps to pull the cell envelope inward. The position of the FtsZ ring is partially determined by the Min system (Loose et al., 2011). The Min system consists of the proteins MinC, MinD, and MinE. MinC inhibits FtsZ ring formation and does so only when bound to MinD. MinD and E form an oscillator that moves MinC, D, and E from one cell pole (bound to the membrane) to the other with a periodicity of about a minute. Because of this oscillator, MinC spends the least time in the mid cell region so that the FtsZ ring can form. An important feature necessary to create oscillations in space is the fact that when MinD is membrane bound, it has a lower diffusion coefficient than when it is free in solution to move to the other cell pole. Hence, diffusion coefficients determine whether the spatiotemporal oscillation can exist.

#### Stable cytoplasmic protein gradients in small cells

A group of proteins can spread within seconds through a cell of several micrometers in length. Because of this, it is not likely that stable protein gradients can form over the length of the cell. However, it has been shown theoretically that protein gradients can form under special circumstances (Lipkow and Odde, 2008). Consider three proteins in a cell: a kinase at one of the cell poles, a phosphatase throughout the cytoplasm, and a substrate protein that can cycle between a phosphorylated and unphosphorylated state. For the system to be able to form a gradient of the substrate protein, the diffusion coefficient of its two states must be different. In a 5 μm long cell, with a kinase rate constant of 10 μm/s (the system is one-dimensional hence the m rather than m^3^), a phosphatase rate constant of 1 s^−1^, and diffusion coefficients of 0.3 μm^2^/s and 10 μm^2^/s for phosphorylated and unphosphorylated forms yields a 10 fold concentration gradient of the substrate protein over the length of the cell. Again, the difference in diffusion coefficients allows the phenomenon to exist.

## New horizons and outstanding questions

Tremendous progress in the determination and understanding of diffusion in a select group of prokaryotes has been made in the last decades. This research has led to the emergence of novel questions, which would lead to improved understanding of the role and importance of diffusion coefficients. In this second part of the review, we will summarize these outstanding questions.

### Consequences of electrostatic steering and ionic strength on diffusion limitation

Earlier we presented the case of the barnase-barstar complex formation. The diffusion limitation that this reaction labors under has been stretched by electrostatic interactions. Yet it is well-known that the on-rate of this particular electrostatic interaction, and others, diminishes with increased ionic strength (Stone et al., 1989; Schreiber and Fersht, 1993; Wallis et al., 1995). This means that organisms with relatively low internal ion concentrations, such as *E. coli* (Shabala et al., 2009), are less affected by diffusion limitation than organisms with high internal ion concentrations, such as *Haloferax volcanii* (Pérez-Fillol and Rodriguez-Vallera, 1986). Does this mean that organisms such as *Hfx. volcanii* are unable to use toxin-antitoxin systems like barnase-barstar? How does this affect transcription factor binding to DNA, or the assembly of ribosomes?

### The effect of temperature on diffusion coefficients

All prokaryotes for which protein diffusion coefficients are known function in a small range of temperatures. How does the diffusion coefficient change if you go from 0 to 100°C ? For proteins in dilute solution we can get an estimate from the Stokes-Einstein equation:

(7) $$D=\frac{k_{B}}{6\pi R}\times \frac{T}{\eta (T)}$$

Here *D* is the diffusion coefficient, *k*_*B*_ is the Boltzmann constant, *R* is the Stokes radius of the protein, *T* is the absolute temperature, and η(*T*) is the viscosity at temperature *T*. We want to know how D changes from 0 to 100°C, and thus need to consider only $\frac{T}{\eta \left(T\right)}$. For 0°C we fill in *T* = 273 K and η(273) = 1.8 × 10^−3^ kg s^−1^m^−1^, for 100°C we fill in *T* = 373 K and η(373) = 0.28 × 10^−3^ kg s^−1^m^−1^. This yields a ~9 fold faster diffusion coefficient at 100 °C. In this calculation we used the viscosity of water. It is unlikely that the Stokes-Einstein equation holds for proteins in the cytoplasm. Firstly, the viscosity is different and not uniform in the cytoplasm, and secondly, and perhaps more importantly, diffusion in cells is probably more affected by excluded volume than by viscosity. It has also been shown that the Stokes-Einstein equation does not hold in the cytoplasm for the relation between diffusion coefficient and Stokes radius (Mika and Poolman, 2011). Nonetheless, the impact of temperature on the diffusion coefficient in cells still needs to be experimentally tested. If there is an increase in diffusion coefficient with temperature, which seems likely, we can make the (conditional) prediction that cells at higher temperatures could have higher cytoplasmic concentrations of macromolecules before essential processes get diffusion limited.

### Direct measurements of diffusion limitation

All examples of diffusion limitation discussed above are based on indirect observations, and rely heavily on modeling (parameters). It would be helpful to have a method for directly determining the diffusion limitation of various processes. That is to vary the diffusion coefficient of one of the actors in the process and then observing whether the rate of the process changes. This is difficult to do because changing the diffusion coefficient can also change other aspects of the cell. Take the example of an osmotic shock which indeed changes the diffusion coefficient (Konopka et al., 2009; Mika et al., 2014), but firstly it does so for all big molecules, secondly it increases ion concentrations of the cytoplasm, and thirdly it increases the excluded volume and therefore affects rates and equilibria of all kinds of processes.

### A note on the use of $\text{d}=\;\sqrt{2\text{nDt}}$

This equation indicates the distance over which a process can act in a given timeframe. Yet this reflects an ensemble of molecules and thus ignores the key characteristic of diffusion: variation of diffusion times for individual proteins. A cell could exploit this variation by using more proteins to send a signal. If you need concentration x at point A for a signal to be effective, you could increase the rate by having more signaling proteins start at point B. It would be interesting to see if this principle could in part explain, for example, the concentrations of two component signaling systems (Capra and Laub, 2012) in the membranes of bacteria.

### Cell size and diffusion length scales

The enormous panoply of prokaryotic species has within itself also a great range of cell sizes. With on the smaller end the Archaeon *Thermodiscus*, with a volume of 3 × 10^−3^ μm^3^, and the bacterium *Mycoplasma pneumoniae*, 5 × 10^−3^ μm^3^. On the larger end we have the bacteria *Epulopiscium fishelsoni*, 3 × 10^6^ μm^3^, and *Thiomargarita namibiensis*, 2 × 10^8^ μm^3^ (Schulz and Jorgensen, 2001). Somewhat counterintuitively both small and large sized could pose challenges for diffusion. For large size, the challenge is obvious; nutrients have to reach parts of the cell from outside of the cell, and proteins have to reach parts of the cell from the chromosome (via mRNA). In *Epulopiscium fishelsoni* and *Thiomargarita namibiensis* this appears to be solved by having many chromosomes, and having them packed against the membrane of the cell. The challenge for the small cells derives from their DNA. *E. coli* has 4.6 Mbp of DNA in a single chromosome (Blattner et al., 1997) and has a volume of about 1 μm^3^ (Taheri-Araghi et al., 2015); *Mycoplasma genitalium* has 0.58 Mbp of DNA (Fraser et al., 1995) and has a volume of about 0.01 μm^3^ (Taylor-Robinson, 1995) (here we assume spherical shape for *M. genitalium*, in reality the cells are pear shaped). The chromosome copy number in *E. coli* depends on growth rate (Stokke et al., 2012), as does its volume (Taheri-Araghi et al., 2015). For the following, we are assuming that the chromosome copy numbers are the same for *E. coli* and *M. genitalium*. The *M. genitalium* volume is 100 times smaller than that of *E. coli*, whereas its genome is only 8 times smaller; leading to a 12.5 times higher concentration of DNA. In *E. coli* the DNA constitutes 3.1% of dry weight, compared to 55% for protein and 20.4% for RNA (Phillips et al., 2009). DNA makes up 3.9% of the *M. genitalium* macromolecules. Multiplying 3.9% by 12.5 gives 49% (that is an extra 45%), so if the protein and RNA content is still the same, we have 1.45 times the amount of macromolecule in *M. genitalium* than in *E. coli*. The consequence that this (potential) difference in volume exclusion has on diffusion coefficients is unclear. For example, when excluded volume is altered by osmotic shocks the effect on the diffusion coefficient appears to be very different in *E. coli* than in *L. lactis* (see Figure 3F). Of course, the distance between any point in the cytoplasm and the outside of the cell is smaller in *M. genitalium* than in *E. coli*, and therefore diffusion is more effective in delivering molecules. However, this distance benefit (in travel time) scales only with the power two (here 21-fold; see Equation 3), whereas the increase in DNA excluded volume scales with the power three (here 100-fold). No studies of diffusion coefficient in prokaryotes have looked at its variation, or lack thereof, along the cell size axis.

The travel distance of a molecule from the membrane to a location in the cytoplasm can be quantified with a characteristic value. The average distance of a point in the cytoplasm to the cell membrane is somewhat less than half the radius. Other distances to consider are for example the average distance between a gene and the membrane or a point in the cytoplasm; the average distance between ternary complex and ribosomes; or the average distance between some position in the cytoplasm and the tip of the stalk of *C. crescentus* (Young, 2006). All these various distances, and the travel times associated with them could be limiting for some process. Consider a *Thiovulum majus* cell that has a diameter of 18 μm (Schulz and Jorgensen, 2001): If the limiting factor was the distance from a gene to a location in the cytoplasm, *T. majus* could just increase its number of chromosomes. Many bacteria are known to have increased numbers of chromosomes (Pecoraro et al., 2011), which would be inconsequential if transport from cell membrane to a point in the cytoplasm is important. Hence, the characteristic distances should be taken into account when dealing with diffusion limitation in prokaryotes.

### Diffusion limitation and membrane proteins

In *E. coli* both plasma- and outer membrane proteins diffuse with much lower diffusion coefficients than cytoplasmic proteins. The same is seen for plasma membrane proteins in *L. lactis* and *C. crescentus* (Table 1). It is unclear if this leads to more diffusion limitation in membrane processes than in cytoplasmic ones. Unlike cytoplasmic proteins membrane proteins can rotate only along one axis which makes it easier for interaction interfaces to find one another. This rotational effect can lower the dissociation constant for a dimerization reaction by orders of magnitude (Grasberger et al., 1986). A similar effect probably also occurs for rates.

### The importance of diffusion coefficients in a cellular context

The rate of a reaction depends on both the concentration of reactants and the on-rate constant (Equation 4). Thus, for diffusion-limited reactions, the rate can be tuned by changing either the concentration or the diffusion coefficient. This means that slower proteins can increase their copy number to have the same interaction rate as smaller proteins. For example, the association rate of ternary complex with ribosomes is capped by limitations on the amount of ternary complex that can be made by a cell, before other processes are adversely affected. Hence, when a process requires the assembly of many proteins, such as ternary complex supplying the amino acids to the ribosome for use in translation, the impact of increasing copy number to increase association rate is tremendous. On the other hand, a change in the association rate for transcription factor binding to a site on the DNA, which needs only one copy (if there is one target site), can be done without much cost. For each protein in the cell one may ask to what degree its copy number is determined by association rate.

From the foregoing paragraph we are led into another question. Is it possible for a cell to have no diffusion limitation? Say we have x amount of protein molecules in a cell and no reaction is diffusion limited. With more protein molecules the cell is able to do more things and, for example, grow faster. So you would expect there to be evolutionary pressure to increase the amount of molecules in cells, and in so doing use up the free, inconsequential, space along the diffusion coefficient axis. Increasing the amount of protein molecules in a cell would continue up to the point that some reactions start to become limiting. This is rather similar to the previous discussion on the relation between cell size and protein concentration, but looked at from a different angle. If true, this means that there will always be diffusion limitation in cells. We can also turn the argument around and ask whether it is possible to have more than one process diffusion limited.

If it would be beneficial to increase the rate of all reactions in a cell, why not make them all steered by electrostatic interactions like the protein interaction pair, barnase-barstar? First, one cannot always change a protein's surface because it could affect its function directly or its stability. Secondly, is it even possible to make electrostatic interactions specific enough so that steering could be done independently for a thousand different interactions? Here the cellular context provides limitations on protein diffusion limited reactions.

### Diffusion may affect different parts of cells differently

Any cell consists of a great number of interlocking and overlapping processes. Protein folding, protein-protein binding, nutrient transport to the cytoplasm, transcription factor binding, structuring the nucleoid, inserting membrane proteins, formation of the Z-ring, Min system cycling, chromosome segregation, cell size maintenance, converting the proteome in response to environmental stress, cell cycle time, etc. For each of these processes we can ask whether they are affected, either in rate or in functional form, by the diffusion coefficients of their constituent proteins. There are bound to be differences between processes in their susceptibility to diffusion changes. Cell cycle time is dependent on the diffusion coefficient of the ternary complex, whereas the cycling rate of the Min system is independent of the cytoplasmic diffusion coefficients of the Min proteins. Processes that require bigger proteins may suffer more from diffusion limitation than processes with small proteins (see Figure 3B). Objects that have a size in the tens of nanometers may also experience other types of mobility (Parry et al., 2014), and processes involving them may thus also be affected. Whether a protein is folded or disordered also seems to have an effect on its diffusion coefficient, with (unexpectedly) a disordered protein diffusing faster than a folded protein in the presence of artificial crowders (Wang et al., 2012). Something discussed earlier relates to the different ranges over which diffusion occurs: translation happens at many places in the cytoplasm with shorter distances between ternary complex and ribosome than, for example, for a two component signaling system that needs to cross the distance between the membrane and a site on the DNA. Different processes are made up of such basic elements in different proportions and may thus be differently affected by changes in diffusion coefficient. Changes in diffusion coefficients can happen in real life situations for example after an osmotic upshift that reduces the cytoplasmic water content. These events could be transient as the uptake of for example K^+^ and compatible solutes counteracts the osmotic imbalance and restores the cell volume (Wood, 2011). To know what the impact of an osmotic upshift is we have to know which processes are vulnerable to a reduction in diffusion coefficient. More generally, we can ask for each process by how many fold the diffusion coefficient needs to go down before this process becomes diffusion limited.

### The reach of diffusion limitation

Cellular processes are layered: (i) The association rate of ternary complex binding to a ribosome is involved in the time of incorporation of a single amino acid into a polypeptide chain (chain elongation); (ii) the rate of chain elongation figures in the rate of protein production; (iii) this in turn determines the rate of accumulation of biomass and cell volume growth, and (iv) together with other processes this sets the cell cycle time. At each layer, the diffusion limitation that sets the rate of a reaction could lose its significance by a slower process in higher layers. How far a diffusion-limited reaction is affecting processes in higher layers is the reach of the diffusion limitation, which should be considered when determining the importance of a diffusion limited protein-protein interactions.

## Conclusion

Protein diffusion coefficients have been determined *in vitro* and in the prokaryotes *E. coli, L. lactis, C. crescentus, P. aeruginosa, Hfx. volcanii*, and others. *E. coli* is the best-studied prokaryote by far. The *in vivo* protein diffusion coefficients have been measured in the cytoplasm, periplasm, plasma membrane, and outer membrane. Various parameters of both proteins and their environment have been compared systematically to the diffusion coefficient, such as protein size, protein surface charge, cytoplasmic ionic strength, and level of excluded volume both *in vitro* and in the cytoplasm. Multiple studies have also been carried out on the importance of the diffusion coefficient in the context of protein toxins, translation, and the level of excluded volume in cells. Yet despite these achievements, the role of diffusion coefficients in prokaryotic cells is still murky. In the future we may look, among other things, into the relation between diffusion coefficient, excluded volume, and temperature; the relation between diffusion coefficient, excluded volume, and cell size; the effect of different diffusion length scales on the impact of diffusion coefficients on various physiological processes; and the complex relation between reaction rates, diffusion coefficients, and protein concentrations. We may also want to try and determine diffusion limitation of processes directly by altering the diffusion coefficient of particular proteins and monitoring the rate of whatever process these proteins function in.

## Author contributions

All authors listed have made substantial, direct, and intellectual contribution to the work and approved it for publication.

### Conflict of interest statement

The authors declare that the research was conducted in the absence of any commercial or financial relationships that could be construed as a potential conflict of interest.
